# Healthcare professionals’ perspectives and/or experiences of digital mental health tools in clinical practice: a systematic review and thematic synthesis

**DOI:** 10.3389/fpsyt.2026.1743614

**Published:** 2026-04-15

**Authors:** Claudia Martin, Emily Eisner, Anja Wittkowski

**Affiliations:** 1 Division of Psychology and Mental Health, School of Health Sciences, The University of Manchester, Manchester, United Kingdom; 2 Greater Manchester Mental Health National Health Service Foundation Trust, Manchester, United Kingdom; 3 Manchester Academic Health Science Centre, Manchester, United Kingdom; 4 The Perinatal Mental Health and Parenting (PRIME) Research Unit, at Greater Manchester Mental Health National Health Service Foundation Trust, Manchester, United Kingdom

**Keywords:** barriers, digital interventions, facilitators, implementation, staff views

## Abstract

**Background:**

Mental health difficulties are highly prevalent worldwide. Digital mental health tools (DMHTs) have been developed to increase accessibility to mental healthcare for people who may struggle to access care due to cost, location or stigma. As the views of stakeholders are important in understanding the potential barriers to and facilitators of DMHT implementation, the aims of this review were to critically appraise and synthesise qualitative findings relating to the perceptions and/or experiences of healthcare professionals (HCPs) on the use of digital mental health tools in clinical practice.

**Method:**

A systematic search of mixed-method and qualitative studies was performed using five databases. Eligible studies were quality-assessed. Data were analysed using inductive thematic synthesis.

**Results:**

Fifteen studies were identified and reviewed. Four main themes (alongside eight subthemes) were developed from the data of 604 HCPs: *1) DMHTs should augment – not replace – face-to-face clinical care; 2) Considerations and caveats to use in clinical practice; 3) Using DMHTs to enhance clinical care;* and *4) Perceived barriers and concerns.*

**Conclusion:**

HCPs strongly endorsed the view that DMHTs offer increased access to care, however, concerns about their therapeutic quality, risk management, and workload burden persist. Context-sensitive implementation and proper infrastructure are essential for successful integration into mental health services.

**Systematic review registration:**

https://www.crd.york.ac.uk/prospero/, identifier CRD42020188879.

## Introduction

Mental health problems are increasingly recognised as a leading cause of disease burden worldwide (1, 2). As of 2025, it is estimated that one in four people will experience a mental health problem in a given year, with the overall lifetime prevalence substantially higher (3, 4). Whilst there are efficacious treatments available, such as medications and talking therapies (5, 6), it is estimated that there are over two million people on the waitlist for mental health treatment in the National Health Service (NHS) in the United Kingdom (UK) (7). In the UK, the socioeconomic cost of untreated mental health problems is great, with an estimated total loss of £300bn *–* double the annual NHS budget of £150bn *–* due to mental ill health in 2022 alone (8). Moreover, untreated mental health problems are associated with high mortality rates due to increased risk of chronic diseases, such as heart disease, diabetes and high blood pressure (9, 10). There is also a threefold risk of self-harm and suicide in these individuals compared to those who receive treatment (11, 12).

There has been global investment to improve access to mental health services, with mental health declared a human right, essential to the development of all countries (13). In the UK specifically, improving access to high quality mental health services *-* including increasing access to digital mental health support *-* are key priorities in the NHS Long Term Plan (14). Furthermore, the rapid digitalisation of mental health services during the COVID-19 pandemic has highlighted a need for technology-delivered interventions and has propelled the development of digital mental health tools (DMHTs) in recent years (15, 16).

DMHTs are defined as any digital platform or app designed to support the prevention, assessment, intervention or management of mental health problems (17). DMHTs include but are not limited to mobile phone apps, online programmes and wearable devices which offer services like mood tracking (18). Previous research has demonstrated several benefits of DMHTs in clinical practice, particularly in terms of accessibility and reducing the stigma associated with help-seeking for mental health problems (19, 20). In light of ongoing staff shortages, DMHTs might support larger numbers of patients to access mental health care (21, 22). Nonetheless, the implementation of DMHTs into clinical practice is poor, with less than 50% of new innovations consistently adopted in routine clinical care (23). In their recent review of 150 studies, the barriers and facilitators of user engagement with DMHTs, Eisner et al. (24) highlighted that staff attitudes played a crucial role in influencing successful implementation of these tools.

Previous reviews have explored the views of HCPs on digital health tools in physical health settings; for example, Borges do Nascimento et al. (25) and Wosny et al. (26) reviewed 43 and 17 studies, respectively. In secondary mental health care settings, Rogan et al. (27) reviewed qualitative studies exploring HCP views on passive sensing and artificial intelligence (AI) specifically. However, no published reviews have examined HCPs’ perspectives on and/or experiences of DMHTs more broadly. Although a relevant mixed-method review protocol was registered on PROSPERO in 2020 (ID: CRD42020188879 weblink: PROSPERO), only the protocol itself has been published so far (28). Interestingly, it aimed to explore the attitudes of primary care professionals only. As HCPs’ perceptions and attitudes pose a potential barrier to implementation (24, 25), it is important to understand their perspectives on DMHTs. Thus, this review aimed to thematically synthesise HCPs’ perspectives and/or experiences of DMHTs in clinical practice, in anticipation that our findings might inform policy, research and future implementation strategies.

## Methods

This systematic review with meta-synthesis was conducted in line with the Preferred Reporting Items for Systematic Reviews and Meta-Analysis (PRISMA) guidelines (29). The protocol was registered with PROSPERO on 27/03/2025 (Ref: CRD420251009218; PROSPERO). The formal synthesis process was also guided by the Enhancing Transparency in Reporting the Synthesis of Qualitative Research (ENTREQ) guidelines (30).

### Search strategy

The search strategy was developed using the SPIDER framework (31) which includes Sample, Phenomenon of Interest, Design, Evaluation, and Research type. The SPIDER framework was selected because it offers a structured, flexible approach for conducting qualitative and/or mixed-method systematic reviews (32). The search terms and associated Medical Subject Headings used are illustrated in **Table 1**. Boolean operators such as “AND” and “OR” were utilised to combine terms within each category. The main reviewer conducted searches combining all categories of SPIDER but also searched without the R (research type) to ensure that papers which did not specify their approach were not missed.

**Table 1:** Search terms, limits and search strategy.

	Category	Search terms
1.	S - Sample	healthcare professional* OR health care professional* OR clinician* OR mental health professional* OR staff*
2.	PI – Phenomenon of Interest	digital mental health* OR digital mental health tool* OR digital mental health intervent* OR digital mental health technology* OR mental ehealth* OR mental mHealth*
3.	D – Design	interview* OR focus group OR survey OR case stud*
4.	E - Evaluation	views OR perception* OR attitude* OR experience* OR perspective* OR view* OR opinions*
5.	R – Research Type	qualitative* OR mixed method*
6.	1 AND 2	
7.	3 AND 4	
8.	5 AND 6 AND 7	

Limits: human, peer-reviewed and English language.

A total of five electronic databases were searched: MEDLINE, PSYCINFO (via Ovid), Web of Science, CINAHL (via EBSCO) and PubMed (via the National Library of Medicine). After an initial search was conducted in January 2023 to help refine the search terms, the final search was conducted in March 2025. Google Scholar, reference lists of included studies as well as similar reviews, were also searched (33).

Identified studies and references were imported into Rayyan (34). Duplicates were removed before titles, keywords, and abstracts were assessed for eligibility against the inclusion and exclusion criteria by the first reviewer. A second independent reviewer assessed a sample of 10% (*n* = 404) of the total number of studies identified (*n =* 4040); agreement between reviewers was substantial (99.75%, κ = 0.79), and any discrepancies were resolved through discussion. Next, the main reviewer reviewed the full text of studies that were included following the screening stage.

### Study inclusion and exclusion criteria

Papers were included if they 1) were written in English, 2) included qualitative data from qualitative OR mixed-methods studies with a substantial qualitative component (e.g., studies with an interview component or surveys which allowed for free text box answers), 3) involved HCPs (either mental health professionals or HCPs working in settings with patients who experience mental health problems), 4) focused on HCP views and/or experiences of DMHTs and 5) were published in a peer-reviewed journal (for quality control and credibility purposes). For the purpose of this review, HCPs were defined as individuals who hold a clinical or therapeutic role within a healthcare setting and who are directly involved in the assessment, treatment, or support of patients or service users.

Papers were excluded if they focused on the views of DMHTs from a patient perspective or if they were quantitative studies. Studies which included mixed participant groups (e.g., HCPs, patients and/or carers together as one sample) were also excluded *unless* HCP views were clearly reported and analysed separately in the results section. This criterion ensured that our synthesis reflected only the perspectives of HCPs. When data from different participant groups were combined and not clearly distinguished, it was not possible to attribute specific findings to the views of HCPs, thereby reducing the relevance and credibility of the data for the purposes of this review. Due to the emerging field of DMHTs (35) no time limits were placed on publications. An outline of the full inclusion and exclusion criteria is illustrated in **Table 2**.

**Table 2:** Inclusion and exclusion criteria.

	Study parameter	Inclusion criteria	Exclusion criteria
1.	Sample population	HCPs only. Both mental health professionals working in settings with patients OR non mental health HCPs (e.g. primary care/physical health services) who work with service users who experience mental health problems. Studies were not required to include HCP job-roles/titles (although studies which did specify job roles were also included). Inclusion was based on the authors’ descriptions of participants as health or clinical professionals working within healthcare settings.	•Studies which focus on the views of patients/non-HCPs. •Studies which focused on the views of patients, carers and HCPs as part of one sample.
2.	Study focus	Studies which investigated the views and/or experiences of HCPs on the use of DMHTs. DMHTs were defined as any digital platform or tool, such as: •mobile phone apps •online programmes, •wearable devices which offer features such as mood tracking.	Studies which investigated HCPs’ views of digital tools for physical health problems OR when the focus of the study was not on HCPs’ views on telehealth (e.g., the format of online therapy delivery within the context of the COVID-19 pandemic), which was not included in our definition of DMHTs.
3.	Methodology	Qualitative methodologies in terms of design (e.g., interview studies, focus groups) and analysis (e.g., thematic, narrative, content analyses, etc.). Can also include mixed-method studies with a significant qualitative component.	•Quantitative studies. •Mixed-method studies with limited qualitative data (e.g., single-word text box survey responses)
4.	Study type	Peer-reviewed primary research.	Non peer-reviewed research (e.g., unpublished theses, opinion pieces).
5.	Language	Written/translated to English.	Not written/translated to English.

### Methodological quality and risk of bias assessment

The methodological quality/risk of bias of each included study was appraised using the widely used ten-item Critical Appraisal Skills Programme for qualitative research (36). The CASP items are typically rated in a ‘yes’, ‘no’ or ‘partially agree’ format, which can be quite subjective (37). For greater clarity and to aid comparison with other qualitative reviews, a numerical system was used to score each item (No=0, Partially Agree=0.5, Yes=1) and to act as a methodological guide for the reader. Scores were added together, and the overall methodological quality was then categorised as either high (>8-10), moderate (6-8) or low (≤ 5), as done by Harries et al. (33) and Butler et al. (38). A second appraisal of all included studies was undertaken by an independent reviewer, and substantial agreement was achieved between reviewers (86.67%, κ = 0.77). Any discrepancies between scores were resolved through discussion.

### Data extraction and analysis

The text from the ‘results/findings’ and ‘discussion’ sections of included papers were imported into NVivo (39) and analysed using thematic synthesis (40). Thematic synthesis is a flexible method of qualitative synthesis that enables reviewers to identify, analyse and interpret patterns from multiple studies with various types of qualitative data analysis (e.g., thematic, framework and content analysis). Furthermore, thematic synthesis supports researchers to develop a comprehensive understanding of the current research landscape which can guide future research, practice and policy making (41).

The three convergent stages of thematic synthesis (40) were followed. Firstly, the main researcher read through each paper several times, then inductively coded the results and discussion sections line by line. These codes were expanded and added to as the main reviewer systematically coded each individual paper. Secondly, the process of categorising and grouping codes together was undertaken. During this stage, the main reviewer re-examined each section of text associated with said codes to ensure there was consistency of interpretation between codes/papers (42). From this, codes were grouped, and new codes were created to capture the meaning of groups of initial codes. In the third and final stage, the main reviewer analysed the descriptive themes to identify more abstract and interpretive themes. Initially, this was done individually by the main researcher, but these were then reviewed and discussed with the review team to ensure they were plausible, coherent, and accurately derived from the data. In the case of any discrepancies or disagreements as to the final theme structure the other two reviewers would independently analyse the included studies and the whole review team would arrive at a final solution through discussion. All authors agreed on the final thematic synthesis.

### Reflexivity statement

The main reviewer approached the analysis from a critical-realist epistemological perspective (43) which offered a framework to understand and interpret the data. This approach supported them to consider both objective (e.g., existing healthcare systems and settings) and subjective (e.g., personal experiences and perceptions) elements to guide their analysis.

## Results

### Study characteristics

**Figure 1:**
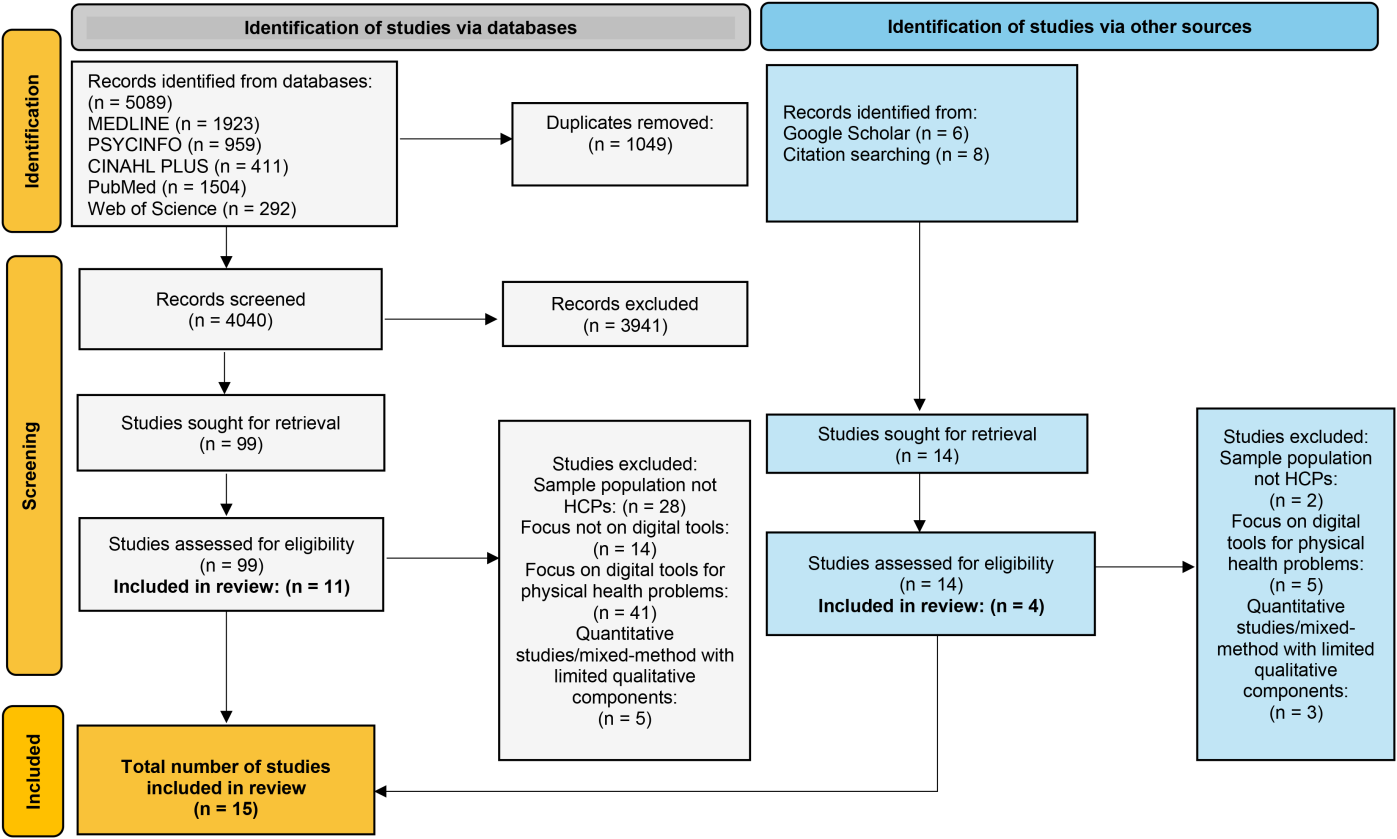
PRISMA flow diagram outlining the systematic process.

The initial search identified 5089 studies, reduced to 4040 after de-duplication. Fourteen articles were added from other sources (citation searching and Google Scholar), resulting in 4054 studies to be screened against eligibility criteria using titles/keywords/abstracts. Of these, 99 full text articles were screened, and 15 studies were included in the review (see **Figure 1**). Although the inclusion criteria permitted both qualitative and mixed-methods studies, all included studies were qualitative in nature, with no eligible mixed-methods studies identified. Included studies were conducted in ten countries, published between 2017 and 2024, and reported on 604 HCPs’ perspectives and experiences of digital mental health tools in clinical practice (see **Table 3**). Most studies (*n* = 13) detailed the professions of their participants, although two studies did not specify this information (44, 54). Sample sizes ranged from nine to 350 participants. Three studies detailed a specific DMHT as a focus of discussion, whereas nine discussed DMHTs more broadly. Participants’ area of work (e.g., healthcare setting), described in 13 studies, were within specialist mental health services (*n* = 8), primary care (*n* = 1) or across both settings (*n* = 4). Specific details of the client populations they served, described in nine studies, were adult only (*n* = 5), children and young people only (*n* = 1), and all ages (child, adult and older adult) (*n* = 3) respectively. Qualitative data were derived from semi-structured interviews (*n* = 11), focus groups (*n* = 3), and an open-ended cross-sectional survey (*n* = 1). Studies were analysed using thematic analysis (*n* = 12), content analysis (*n* = 2), and framework analysis (*n* = 1).

**Table 3:** Characteristics of included studies presented in reverse chronological order.

	Study authors, year and location	Study aim(s)	Sample description	Description of digital tool	Service and/or client group	Data collection and analysis	Main themes
1	Moitra et al. (44) 2024 USA	to characterise stakeholders’ perspectives on potential facilitators and barriers to care, and to seek input regarding the appropriateness and acceptability of mHealth in their treatment setting	18 HCPs (job roles not specified)	mobile app (name not specified – still in development)	specialist mental health services; adults with schizophrenia spectrum conditions	semi-structured interviews; applied thematic analysis (45)	1. adherence challenges 2. role of mobile technology in patient care 3. clinical professionals’ receptiveness to adjunctive mHealth services 4. costs related to implementation of mHealth services.
2	Hildebrand et al. (46) 2024 Germany	to explore psychotherapists’ perspectives on opportunities for improvement, facilitating conditions, and barriers to using DMHTs	350 HCPs, including: cognitive behavioural therapists (*n=*303), psychodynamic therapists (*n=*30), analytical therapists (*n=*9), systemic therapists (*n=*3), other (*n=*5)	not specified	across settings; patient group not specified	Open-ended cross-sectional survey; thematic analysis (47)	1. applicability 2. treatment resources 3. technology 4. perceived risks and barriers
3	Rogan et al. (27) 2024 UK	to explore mental health professionals’ views on using digital devices to passively collect data and apply machine learning in mental health care, as well as the potential barriers and facilitators to their implementation in practice	15 HCPs, including: psychologists (*n=*4), psychiatry trainees (*n=*2), consultant psychiatrists (*n=*2), mental health nurses (*n=*5), occupational therapist (*n=*1), mental health practitioner (*n=*1)	not specified	specialist mental health services; adults	semi-structured interviews; thematic analysis (47)	1. positives and negatives of digital devices, passive sensing and machine learning methods in mental healthcare 2. barriers to use in clinical practice 3. facilitators for clinicians
4	Bassi et al. (48) 2024 Canada	to explore mental health professionals’ perceptions of the barriers and facilitators that may influence their utilization of digital MH-enabled measurement-based care	10 HCPs, including: counsellors/therapists (*n=*50), psychologists (*n=*13), administrators (*n=*5), medical staff (*n=*22), unknown (*n=*13)	measurement-based care (MBC) application “Innnowell Platform”	specialist mental health services; youth defined as 16–24 years old	focus groups; thematic analysis (47)	1. cultural stigma, family apprehension about mental health care, and parental access, including consent and access 2. perceptions of increased responsibility and liability for a youth in crisis 3. perception that some psychiatric and neurodevelopmental disorders in youth are not amenable to dMH 4. youth readiness to engage with dMH 5. youth with mild mental health concerns are the best fit for dMH 6. youth motivated to report dynamic change in mental health 7. youth proficiency and preferences for dMH and MBC
5	Mayer et al. (49) 2024 Germany	to gain insights into the experiences, perspectives, and expectations of mental health professionals for EDs regarding DMHIs and to identify requirements for future integration of DMHIs into routine care	24 HCPs, including: psychologists (*n=*14), social worker (*n=*1), medical doctors (*n=*7), paedology (*n=*2)	not specified	inpatient and outpatient eating disorders service; adult and child.	semi-structured interviews; thematic analysis (47)	1. experiences with DMHIs 2. advantages and chances 3. disadvantages and boundaries 4. desired functions and properties 5. target groups of DMHIs 6. general conditions and requirements 7. requirements for integration of DMHIs into other areas of healthcare spectrum
6	Scott et al (50) 2023 Australia	to explore psychologists perceptions and experiences of digital mental health in practice	10 clinical psychologists (*n* = 10)	not specified	across settings; all age	semi-structured interviews; thematic analysis (47)	1. attitudes towards digital mental health 2. use within routine practice 3. perspectives on an effective model for implementation.
7	Lukka et al. (51) 2023 Finland	to create a contextual understanding of how MHPs use different digital tools in clinical client practice and what characterizes the use across tools	19 HCPs, including: occupational therapist (*n=*1), nurses (*n=*7), psychologists (*n=*11)	not specified	specialist mental health services; all age	semi-structured interviews; thematic analysis (47)	1. HCPs use complementary channels in client communication 2. the evaluation of clients is being digitized 3. HCPs support therapeutic change with digital materials 4. digital tool use is negotiated in client interaction 5. autonomy and contexts diversify the MHP ‘Digital Toolbox’ 6. existing practices invite incremental developments
8	Mendes-Santos et al. (52) 2022 Portugal	to explore factors affecting perspectives and practices of MHPs regarding the implementation of digital mental health	13 HCPs, including: psychiatrists (*n=*2), psychologists (*n=*11)	not specified	across care settings; client population not specified	semi-structured interviews; thematic analysis (47)	1. indication evaluation 2. therapeutic contract negotiation 3. digital psychological assessment 4. technology setup and management 5. intervention delivery and follow up
9	Melia et al. (53) 2021 Ireland	what are the experiences of MHPs engaged in the use of mobile apps as part of their practise? what are the drivers and barriers and best theoretical model to account for MHPs engagement in these technologies?	15 HCPs, including: psychiatrists (*n=*2*)*, psychologists (*n=*13)	not specified	across care settings; all age	semi-structured interviews; thematic analysis (47)	1. accessibility 2. transitional object 3. integration 4. trust
10	Silfee et al. (54) 2021 USA	to assess providers’ views on the feasibility and acceptability of delivering a cognitive behavioural therapy (CBT) based mobile app in multiple care settings	19 physical and behavioural health providers (job roles not specified)	CBT based mobile phone app ‘RxWell’	primary care; client population not specified	semi-structured interviews; content analysis (55)	1. benefits and complexities of incorporating the app into the provider’s treatment toolkit 2. factors that influence both provider and patient engagement 3. challenges and opportunities with current provider monitoring capabilities 4. potential for sustaining app referrals in clinical setting
11	Pithara et al. (56) 2020 UK	to examine mental health care providers’ views of and experiences with the CPT during the pilot implementation phase and identify factors influencing its implementation.	20 HCPs, including: mental health support workers, peer support workers, psychiatrists, occupational therapists, community psychiatric nurses, and social workers (*n* not specified)	mobile based app “Care Pathway Tool”	specialist mental health services; adults	semi-structured interviews; thematic analysis (47)	1. innovation domain 2. outer setting domain 3. inner setting domain 4. individuals domain 5. implementation process domain
12	Bucci et al. (57) 2019 UK	to explore staff views regarding the utility and appropriateness of using digital tools in the healthcare pathway for people accessing specialist secondary care mental health services	48 HCPs, including: care coordinators (*n* = 10); clinical psychologists (*n* = 8), mental health practitioners (*n* = 5), team managers (*n* = 5), support, time, and recovery (STR) workers (*n* = 5), community psychiatric nurses (*n* = 4), social workers (*n* = 4), psychiatrists (*n* = 4), researchers (*n* = 2), and a team secretary (*n* = 1)	not specified	specialist mental health services; adults	focus groups; framework analysis (58)	1. perceived barriers to adopting digital health interventions 2. acceptability of digital health interventions for people accessing early intervention for psychosis services 3. data security, safety and risk 4. relationships
13	Feijt et al. (59) 2018 Netherlands	to understand drivers and barriers for psychologists in adopting eMH tools	12 HCPs, including: clinical psychologists (*n=*12)	not specified	setting and client population not specified	semi-structured interviews; thematic analysis (47)	1. general characteristics of eMental Health 2. perceived drivers 3. perceived barriers 4. contextual factors of daily clinical practice
14	Krog et al. (60) 2018 Denmark	to explore barriers and facilitators to using a web-based version of the Major Depression Inventory (eMDI) in general practice	9 HCPs, including: general practitioners (*n=*9)	web version of the Major Depression Inventory ‘eMDI’	setting and client population not specified	semi-structured interviews; thematic analysis (47)	1. psychological capability 2. social opportunity 3. physical opportunity 4. reflective motivation 5. automatic motivation
15	Berry et al. (61) 2017 UK	to explore mental health care staff experiences of clients with severe mental health problems engaging with the Internet and mobile phones to self-manage their mental health and their views toward these behaviours	20 HCPs, including: psychologists (*n=*7), mental health nurses (*n=*3), support worker (*n=*1), occupational therapists (*n=*2), practice nurse (*n=*1), team leader (*n=*1)	not specified	specialist mental health services; adults	focus groups; thematic analysis (47)	1. digital health interventions could increase access to mental health support options, but may perpetuate the digital divide 2. staff have conflicting views about the pros and cons of using web-based resources and digital health interventions to manage mental health 3. digital health interventions’ impact on staff roles and responsibilities 4. digital health interventions should be used to enhance, not replace, face-to-face support

ED, eating disorder; DMHT, digital mental health tool; MHP, mental health professional; HCP, healthcare professional; CBT, cognitive behaviour therapy; STR Worker, support time recovery worker; GP, general practitioner; dMH/eMH, digital mental health; mHealth, mobile health. Methodological quality of included studies.

All 15 studies were assessed as being of high methodological quality (see **Table 4**). Eight studies received the highest methodological quality rating of 10/10 (27, 48, 49, 52, 53, 57, 60, 61). Of the remaining seven studies, one aspect (adequate consideration of the researcher-participant relationship) was most often not expressed well. Almost all studies adequately considered ethical issues. One study did not report these clearly (54) and this study received the lowest overall quality rating of 8/10. No studies were excluded from this systematic review.

**Table 4:** Overview of the methodological quality assessment of included studies using the CASP.

	Study Authors and Year	Clear statement of the aims of the research?	Is a qualitative method appropriate?	Was the research design appropriate to address the aims of the research?	Was the recruitment strategy appropriate to the aims of the research?	Was data collected in a way that addressed the research issue?	Has the relationship between the researcher and participants been adequately considered?	Have ethical issues been taken into account?	Was the data analysis sufficiently rigorous?	Is there a clear statement of findings?	How valuable is the research?	Overall appraisal score:
**1**	Moitra et al. (44)	Y (1)	Y (1)	Y (1)	Y (1)	Y (1)	N (0)	Y (1)	Y (1)	Y (1)	Y (1)	High (9)
**2**	Hildebrand et al. (46)	Y (1)	Y (1)	Y (1)	Y (1)	Y (1)	N (0)	Y (1)	Y (1)	Y (1)	Y (1)	High (9)
**3**	Rogan et al. (27)	Y (1)	Y (1)	Y (1)	Y (1)	Y (1)	Y (1)	Y (1)	Y (1)	Y (1)	Y (1)	High (10)
**4**	Bassi et al. (48)	Y (1)	Y (1)	Y (1)	Y (1)	Y (1)	Y (1)	Y (1)	Y (1)	Y (1)	Y (1)	High (10)
**5**	Mayer et al. (49)	Y (1)	Y (1)	Y (1)	Y (1)	Y (1)	N (0)	Y (1)	Y (1)	Y (1)	Y (1)	High (9)
**6**	Scott et al. (50)	Y (1)	Y (1)	Y (1)	Y (1)	Y (1)	N (0)	Y (1)	Y (1)	Y (1)	Y (1)	High (9)
**7**	Lukka et al. (51)	Y (1)	Y (1)	Y (1)	Y (1)	Y (1)	Y (1)	Y (1)	Y (1)	Y (1)	Y (1)	High (10)
**8**	Mendes-Santos et al. (52)	Y (1)	Y (1)	Y (1)	Y (1)	Y (1)	Y (1)	Y (1)	Y (1)	Y (1)	Y (1)	High (10)
**9**	Melia et al. (53)	Y (1)	Y (1)	Y (1)	Y (1)	Y (1)	Y (1)	Y (1)	Y (1)	Y (1)	Y (1)	High (10)
**10**	Silfee et al. (54)	Y (1)	Y (1)	Y (1)	Y (1)	Y (1)	N (0)	N (0)	Y (1)	Y (1)	Y (1)	High (8)
**11**	Pithara et al. (56)	Y (1)	Y (1)	Y (1)	Y (1)	Y (1)	N (0)	Y (1)	Y (1)	Y (1)	Y (1)	High (9)
**12**	Bucci et al. (57)	Y (1)	Y (1)	Y (1)	Y (1)	Y (1)	Y (1)	Y (1)	Y (1)	Y (1)	Y (1)	High (10)
**13**	Feijt et al. (59)	Y (1)	Y (1)	Y (1)	Y (1)	Y (1)	N (0)	Y (1)	Y (1)	Y (1)	Y (1)	High (9)
**14**	Krog et al. (60)	Y (1)	Y (1)	Y (1)	Y (1)	Y (1)	Y (1)	Y (1)	Y (1)	Y (1)	Y (1)	High (10)
**15**	Berry et al. (61)	Y (1)	Y (1)	Y (1)	Y (1)	Y (1)	Y (1)	Y (1)	Y (1)	Y (1)	Y (1)	High (10)
% of studies meeting each item of the CASP:		100%	100%	100%	100%	100%	53.3%	93.3%	100%	100%	100%	

An answer of Y (yes) means that the study fully met the criteria for that item. An answer of N (no) means that the item did not meet the criteria for that item. Thematic synthesis.Green means yes or 1, red means no or 0. Yellow indicates percentage agreement for each CASP question.

Four main themes (and eight associated subthemes) were developed from the data, illustrating HCPs’ perceptions and experiences of DMHTs in clinical practice: *1) DMHTs should augment - not replace – face-to-face clinical care, 2) Considerations and caveats to use in clinical practice* (with two sub-themes), *3) Using DMHTs to enhance clinical care* (with two sub-themes), and *4) Perceived barriers and concerns* (with two sub-themes). Illustrative quotes are provided within the text in italics. A matrix of theme representation across the 15 studies are illustrated in **Table 5**.

**Table 5:** Matrix of theme representation within the 15 included studies.

Study authors and year:		Theme 1: DMHTs should augment - not replace – face-to face-clinical care	Theme 2: Service user considerations and caveats to use in clinical practice		Theme 3: Using DMHTs to enhance clinical care		Theme 4: Perceived barriers and concerns regarding the use of DMHTs in clinical practice	
			2.1 Service user characteristics	2.2 Clinical risk considerations	3.1 Facilitators of engagement with DMHTs for HCPs	3.2 Perceived benefits of DMHTs for service users	4.1Workload burden as a barrier to implementation	4.2 Risk of harm to service users
1	Moitra et al. (44)	–	–	✓	✓	✓	✓	✓
2	Hildebrand et al. (46)	✓	✓	✓	–	–	✓	✓
3	Rogan et al. (27)	–	✓	✓	✓	✓	✓	✓
4	Bassi et al. (48)	–	✓	✓	✓	–	✓	✓
5	Mayer et al. (49)	✓	✓	✓	✓	✓	✓	✓
6	Scott et al. (50)	✓	✓	✓	–	✓	✓	✓
7	Lukka et al. (51)	✓	✓	✓	–	✓	–	–
8	Mendes-Santos et al. (52)	✓	–	✓	✓	✓	✓	✓
9	Melia et al. (53)	✓	–	–	✓	✓	✓	✓
10	Silfee et al. (54)	✓	✓	✓	✓	✓	–	–
11	Pithara et al. (56)	✓	✓	✓	–	✓	✓	–
12	Bucci et al. (57)	✓	✓	✓	✓	✓	✓	✓
13	Feijt et al. (59)	✓	✓	✓	✓	✓	✓	✓
14	Krog et al. (60)	✓	✓	✓	✓	✓	✓	–
15	Berry et al. (61)	✓	✓	✓	✓	✓	✓	✓

### Line of argument synthesis

**Figure 2:**
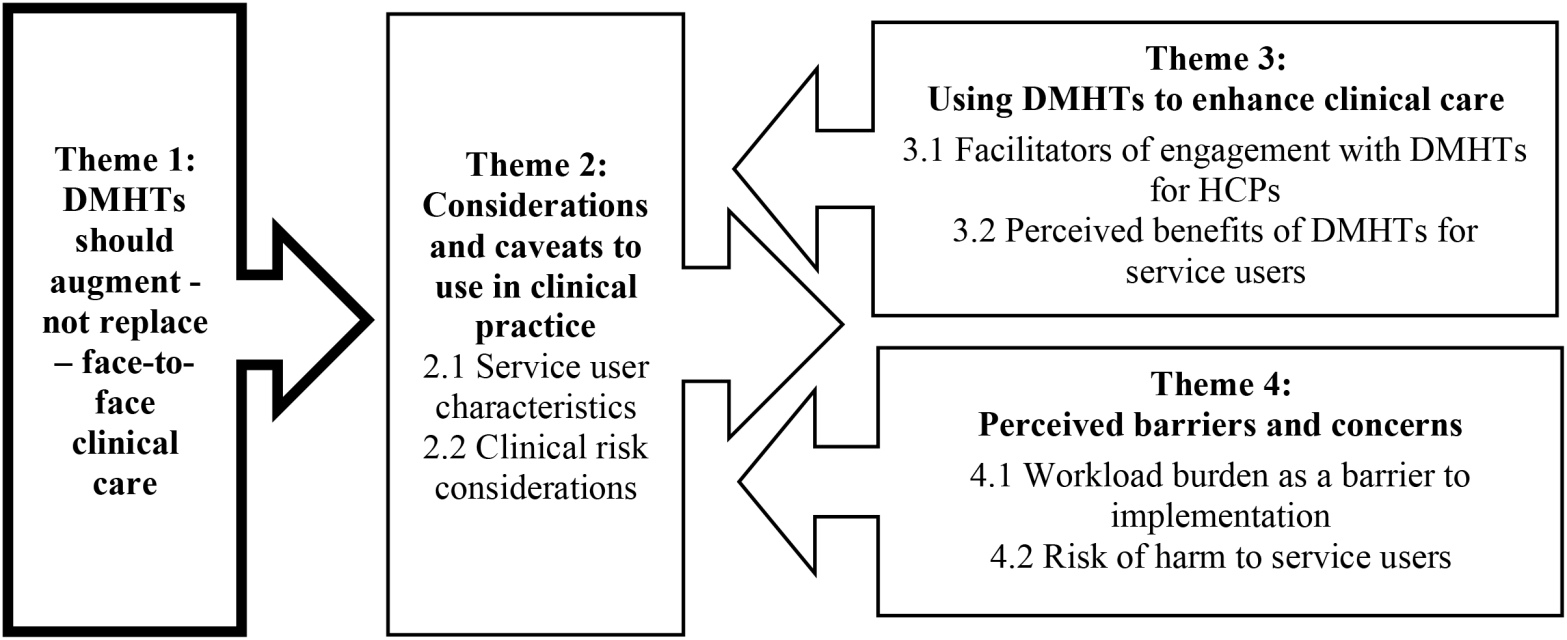
Line of argument synthesis model.

Through the analysis process, a line of argument (LOA) synthesis (62) emerged. To aid the reader, the relationship between the main themes and subthemes are depicted in **Figure 2**, using the LOA synthesis model. The model reflects HCPs position that DMHTs should be used to augment rather than replace face-to-face clinical care, forming the central organising concept. This is shaped by contextual factors such as service user characteristics (e.g., age, digital literacy) and clinical risk considerations (e.g., psychiatric diagnoses, presence of suicidality), both of which influence HCP views of the appropriateness, and acceptability of DMHTs. This also feeds into how DMHTs might be used in practice, with facilitators for HCPs (e.g., usefulness, accessibility) and perceived benefits for service users (e.g., engaging hard to reach populations, service user empowerment) emphasised. The model also captures the dual nature of engagement, with barriers for HCPs (e.g., workload implications) and safety concerns for service users (e.g., risk of pathologising) which might inhibit DMHT uptake within services. The following section presents the review findings according to the LOA synthesis.

### Theme 1: DMHTs should augment – not replace – face-to-face clinical care

Theme 1 was characterised by one of the strongest messages arising from the studies, HCPs’ concerns about DMHTs potentially replacing face-to-face clinical care. The view that DMHTs should *“never replace a face-to-face therapy”* (Mayer et al. (49), p. 5) was a common theme across all studies, with DMHTs perceived as *“very depersonalised”* and *“lacking warmth”* (Bucci et al. (57), p. 11). As such, HCPs described the “*indispensability of face-to-face contact”* (Feijt et al. (59), p. 4) for the purpose of psychological evaluation as a way to gain a sense of the *“whole person”* and *“observe and react to nuances in client expressions and behaviour that would otherwise be lost”* (Lukka et al. (51), p. 7). HCPs expressed fear that they would lose their *“clinical sense”* (Participant, Mendes-Santos et al. (52), p. 9) if psychological assessments were conducted digitally. These concerns were amplified for HCPs who worked with vulnerable service users with complex mental health needs (e.g., in secondary mental health services), as described by Participant 31:

*“All of those kinds of non-verbal cues and para-language is lost in electronic communication, and a lot of what we do relies on non-verbal cues and para-language. Saying that they’re fine and they’re still in their bedclothes that they’ve been wearing for three days … IT [information technology] would never tell me that”* (Participant, Bucci et al. (57), p. 9).

A sense of fear that DMHTs could compromise the therapeutic alliance was prevalent across almost all studies. HCPs regarded face-to-face interventions, such as psychotherapy, as a superior mode of delivery for mental health treatment, with a *“real connection between people”* (Participant, Pithara et al. (56), p. 5) viewed by HCPs as a *“prerequisite for change during therapy”* (Mayer et al. (49), p. 6). In all studies, HCPs placed high value on *“the connection and the relationship and feeling comfortable”* (Participant, Scott et al. (49), p. 346), with face-to-face interaction recognised as a conduit to a *“strong therapeutic alliance”* (Rogan et al. (27), p. 11), with the client-therapist relationship acknowledged as *“therapeutic in itself”* (Lukka et al. (51), p. 7). In terms of mental health outcomes, HCPs considered the therapeutic alliance as “*the strongest agent of change”* (Melia et al. (53), p. 7). Preserving face-to-face contact was considered particularly important for certain presentations, such as depression and obsessive-compulsive disorder: “*they all require direct human contact for effective treatment”* (Participant, Hildebrand et al. (46), p. 5). Furthermore, one participant also considered particular limitations of DMHTs for service users presenting with interpersonal difficulties:

*“Personality disorders, for example. I fear that the relational component is missing in order to actually make a change (…) for me, it depends on the severity of the disorder.”* (Participant, Hildebrand et al. (46), p. 5).

Whilst HCPs believed DMHTs should *“never be offered as a replacement to face-to-face support*” (Berry et al. (61), p. 10), they acknowledged that DMHTs might be a useful adjunct to face-to-face contact, as a *“supplement to an actual consultation”* (Krog et al. (60), p. 5).

HCPs identified many uses of DMHTs to support clinical care, such as *“mood and goal monitoring”* (Participant, Melia et al. (53), p. 7), *“helping you to remember to do skills”* (Participant, Silfee et al. (54), p. 6), *“to help identify triggers and patterns”* (Participant, Berry et al. (61), p. 7), and *“monitoring your own symptoms”* (Participant, Moitra et al. (44), p. 214). HCPs perceived the use of DMHTs in this capacity might *“offer better preparation before a consultation”* (Participant, Krog et al. (60), p. 6), with information generated from DMHTs followed up on during face-to-face contact with service users.

### Theme 2: considerations and caveats to use in clinical practice

The second theme and its two subthemes encapsulated HCPs’ views on which service user populations might benefit the most from DMHTs, including their rationale for these perceptions. HCPs also emphasised level of risk as a critical factor when determining whether or not DMHTs were suitable as part of clinical care.

#### Subtheme 2.1: service user characteristics

Across studies, HCPs reflected on the specific characteristics of their service user populations, particularly in relation to their ability to engage with DMHTs. Most HCPs agreed that DMHTs would be most accepted by “*the younger digital native generation”* (Bucci et al. (57), p. 10) who were more accustomed to communications within *“the digital space”* (Mayer et al. (49), p. 5). HCPs described this generation as “*pretty tech savvy”* (Participant, Bassi et al. (48), p. 9), often exceeding HCPs’ own understanding and knowledge of technology: “*they know way more than I do about tech”* (Participant, Bassi et al. (48), p. 9). With regular internet and mobile phone use viewed as the *“norm”* (Berry et al. (61), p. 7) in this population, HCPs expressed confidence that DMHTs might be “*a mechanism to improve access to mental health support”* (Berry et al. (61), p. 7) for these service users.

In contrast, some service users, such as older adults, were perceived as *“less tech-savvy”* (Participant, Silfee et al. (54), p. 6) by HCPs. Many HCPs shared the view that older service users might be less able to engage with DMHTs as part of their care:

*“I fear I’m being a bit stereotypical, but maybe … it’s a big change for maybe an older adult population who never grew up with this kind of technology****”*** (Participant, Rogan et al. (27), p. 9).

This aspect was a particular concern in terms of service delivery, including the impact of time spent supporting service users less familiar with technology *“who find their phone is merely a communication device rather than an all-in-one type of device”* (Participant, Silfee et al. (54), p. 6) to use DMHTs. Nonetheless, HCPs acknowledged that the age of service users should not be exclusive; rather*: “it depends on their level of awareness, where they are at in their recovery as well”* (Participant, Pithara et al. (56), p. 5).

HCPs also considered the *“digital divide”* (Bucci et al. (57), p. 6), such as poverty, disability and illiteracy, as barriers to service user engagement with DMHTs. Ultimately, most HCPs agreed that the decision to use DMHTs should be *“collaborative”* (Participant, Rogan et al. (27), p. 11) between HCPs and service users, with due consideration of *“individual, technological, and contextual factors”* (Mendes-Santos et al. (52), p. 8) at the centre of the decision:

*“I have the overall approach that I offer the client different means, tools, and then they decide.”* (Participant, Lukka et al. (51), p. 10).

#### Subtheme 2.2: clinical risk considerations

HCPs considered the suitability of DMHTs by the severity of service users’ mental health problems. DMHTs were considered most appropriate for service users with *“less severe mental health challenges”* as a *“low-threshold service for prevention”* (Participant, Hildebrand et al. (46), p. 6). The concept of ‘less severe’ was a common theme across studies and defined in several ways, including *“relatively mild psychiatric problems, such as subclinical anxiety”* (Participant, Lukka et al. (51), p. 9), “*those in the mild to moderate range”* (Participant, Silfee et al. (54), p. 6), and those *“in the early stages of illness”* (Participant 4, Mendes-Santos et al. (52), p. 7). DMHTs were also considered valuable if integrated within a stepped care model, as a tool to prevent less severe cases from developing into more serious mental health problems:

*“Perhaps it [DMHTs] can effectively prevent future psychiatric illness”* (Participant 4, Mendes-Santos et al. (52), p.7).

Some HCPs *“valued the opportunity to identify patients at risk for self-harm”* (Moitra et al. (44), p. 214) and recognised that DMHTs could be utilised within services as a way to triage and prioritise face-to-face support for those with more severe mental health problems. For example, services could *“have our therapists focus instead on those that truly need to see them”* (Participant 2, Bassi et al. (48), p. 8), whilst ensuring those considered less severe still have access to some sort of mental health support.

Nonetheless, the majority of HCPs warned that DMHTs should be used with caution or altogether avoided in cases of severe mental health difficulties. Severe mental health difficulties were broadly defined across studies as: “*suicidal crisis or self-harm”* (Mayer et al. (49), p. 5), “*psychosis”* (Rogan et al. (27), p. 10),*”severe behavioural symptoms”* (Silfee et al. (54), p. 8), *“personality disorders”* (Hildebrand et al. (46), p. 6; Bassi et al. (48), p. 7), and *“severely ill patients who suffer from suicidal thoughts”* (Krog et al. (60), p. 6). Specific reservations related to the use of DMHTs in these presentations were related to HCPs’ ability to respond to risk in an appropriate and timely manner, particularly in situations whereby service users might have disclosed *“suicidal thoughts and behaviours outside of regular sessions and work hours”* (Bassi et al. (48), p. 6). Across most studies, HCPs expressed their discomfort with receiving risk information from DMHTs due to *“difficulties in managing crisis episodes from a distance”* (Mendes-Santos et al. (52), p. 8) and being “*informed too late to intervene in time”* (Mayer et al. (49), p. 6). In particular, HCPs were apprehensive about their ability to successfully respond to these risks within the context of their existing clinical duties, raising questions as to *“where my responsibilities lie”* (Participant, Feijt et al. (59), p. 6).

### Theme 3: using DMHTs to enhance clinical care

The third theme, divided into two subthemes, represented the benefits and facilitators of DMHTs perceived by HCPs across studies. The terms *‘benefits’* and *‘facilitators*’ are often used interchangeably within implementation research. For the purpose of this review, *‘benefits’* refer to HCPs’ views on the positive outcomes and/or effects of DMHTs, whilst *‘facilitators’* refer to factors that help and/or support successful implementation from an HCP perspective.

#### Subtheme 3.1: facilitators of engagement with DMHTs for HCPs

HCPs recognised *“practical personal benefits”* of utilising DMHTs as part of their clinical practice, such as an *“increased efficiency in administrative tasks”* (Feijt et al. (59), p. 5). Many HCPs discussed the utility of DMHTs in terms of providing rich contextual information that is *“usually unattainable during in-person appointments”* (Mendes-Santos et al. (52), p. 10). HCPs contemplated if using DMHTs that allow service users to record their experiences immediately might allowed to *“a more ecologically valid assessment of symptoms”* (Participant, Bucci et al. (57), p. 6) when compared to reliance on retrospective self-reports during face-to-face assessments. One HCP described how their experience of using DMHTs supported them to understand the context of their client’s difficulties, which would have otherwise been missed:

*“I completely entered her world (…) I could see the tidiness and untidiness of her little room (…) Often I could see that she was wearing pyjamas (…) she was presenting with depressive symptoms (…) and I was there to watch it like a movie”* (Participant, Mendes-Santos et al. (52), p. 10).

Furthermore, HCPs described DMHTs as “*welcomed as a tool for prioritising time*” (Krog et al. (60), p. 5) and recognised the benefits of being able to gain valuable information about service users’ mental state “*as we go and as they start to decline”* (Participant, Bassi et al. (48), p. 9) in a way that has never before been possible: “*we are capable of following their development over time, which we have neglected before*” (Krog et al. (60), p. 6). HCPs viewed DMHTs as a potential aid to help prioritise those in need of swift support: *“to assess a potential crisis before it happens (…) maybe deal with things quicker rather than they’re waiting and waiting and waiting for their appointment to come up*” (Participant, Bassi et al. (48), p. 8).

#### Subtheme 3.2: perceived benefits for service users

HCPs identified several potential benefits of DMHTs for service users. Many HCPs, across studies, highlighted the potential of DMHTs to engage hard-to-reach service users, such as “*engaging individuals for whom traditional face-to-face mental health services are not appropriate or accessible”* (Melia et al. (53), p. 6), including *“geographically isolated clients, migrants, and clients at risk of contracting infectious diseases”* (Mendes-Santos et al. (52), p. 8). Furthermore, HCPs emphasised that service users were *“not always forthcoming with information”* (Moitra et al. (44), p. 214) and might have different communication preferences than those currently offered by services. HCPs recognised that face-to-face consultations might be *“tough”* to access for some individuals: *“they are not hard-to-reach clients, we have just got hard-to-reach services”* (Participant, Bucci et al. (57), p. 6). HCPs suggested that DMHTs might allow for more *“flexibility for how people want to connect”* (Scott et al. (49), p. 345), thus enabling previously hard-to-reach individuals, *“particularly service users who find direct social interaction challenging, or who have an interest in technology”* (Participant, Melia et al. (53), p. 6), to engage with services.

Several studies also highlighted how *“shame and fear of stigmatisation*” (Mayer et al. (49), p. 7) were commonplace with regard to mental health problems. HCPs identified stigma reduction as a main benefit of DMHTs due to the *“everyday nature of a mobile phone and availability of an app”.* Apps were seen as *“very, very discrete … it just looks like you’re texting or just using a normal app or something”* (Participant, Bucci et al. (57), p. 7), providing opportunities for *“a very suitable, low-threshold facilitator to help-seeking”* (Mayer et al. (49), p. 7) that was *“less intimidating”* (Participant, Moitra et al. (44), p. 214) than traditional methods of communicating distress to care providers.

HCPs also suggested that this might empower service users to take a more active role in their care and treatment: *“there’s the proactive ‘I’ve got some control’ element, which is what we always want for people”* (Participant, Rogan et al. (27), p. 6). Concepts of control and empowerment were described as benefits of DMHTs by service users, according to one HCP, who piloted a mobile app in their clinical service:

*“I’ve had feedback that service users have felt in the centre of the process. [ … ] more in control of their support [ … ] by being part of that process and by having the opportunity to use the tool”* (Participant, Pithara et al. (56), p. 5).

### Theme 4: perceived barriers and concerns

The final theme illustrated the potential barriers and concerns of DMHT use in clinical practice, as perceived by HCPs. *‘Barriers’* (e.g., factors which obstruct DMHT use for HCPs) and *‘concerns’* (e.g., perceived risks of DMHT use) are organised into two subthemes: *workload burden as an implementation barrier* and *risk of harm to service users*.

#### Subtheme 4.1: workload burden as an implementation barrier

Workload concerns were consistently highlighted by HCPs as a major barrier to DMHT use. Virtually all HCPs reported that they were *“constrained in terms of time and availability”* (Moitra et al. (44), p. 214) as well as *“under-resourced and already carrying a huge workload”* (Participant, Bassi et al. (48), p. 6). A sense of apprehension that DMHTs might make HCPs’ work *“more burdensome”* (Participant, Feijt et al. (59), p. 6) was prevalent across studies. Overall, HCPs described themselves as *“too busy”* (Moitra et al. (44), p. 214) to hold an additional responsibility to access and monitor DMHT-generated data, such as mobile app alerts. One HCP described capacity issues during the pilot of a mobile phone app within their service: *“services got very, very swamped with huge numbers of referrals coming in”* (Participant, Pithara et al. (56), p. 5).

As a result, many HCPs expressed doubt in their ability to manage alerts pertaining to risk through DMHTs: ‘*what happens if I get a flag that one of my patients is suicidal? Where does that fit in my day?”* (Participant, Bassi et al. (48), p. 6). HCPs also reflected on further consequences of missed risk, which might arise when service users reached out for help via DMHTs and risk indicators were not acted upon. HCPs across multiple studies spoke candidly about their fear of *“potential liability issues”* (Moitra et al. (44), p. 214) and being held legally responsible in cases of service user suicide. One HCP reflected on how such situations could be scrutinised in court:

*“if there was an indicator that would concern you … if you haven’t seen that and then something happened, how would that stand up in a coroner’s court?”* (Participant, Rogan et al. (27), p. 11).

#### Subtheme 4.2: risk of harm to service users

HCPs expressed concern that DMHTs might unintentionally cause harm to service users by pathologising normal experiences and symptoms. HCPs warned that some service users might tend to *“oversubscribe to certain symptoms”* (Bassi et al. (48), p. 7), whilst others noted that DMHTs might encourage service users to *“unhelpfully dwell on experiences”* (Berry et al. (61), p. 7), highlighting the risk that excessive self-reflection promoted by DMHTs could reinforce rumination. HCPs highlighted that rumination might be particularly problematic with mood tracking features, especially for service users who might already be anxious about their health. HCPs explained that focusing too much on data could *“create a fear of relapse, causing service users to over‐ or misinterpret a natural ebb and flow in mood”* (Rogan et al. (27), p. 10) and that DMHTs might cause some users to interpret objectively benign thoughts or moods through a pathological lens. As a consequence, HCPs warned that DMHTs might create unnecessary concerns for both the service user and their clinical team. Furthermore, for service users with avoidant tendencies, HCPs explained that online formats of care could in fact reinforce maladaptive patterns of behaviour:

*“if it’s always online and one of the issues is that they’re not able to be face-to-face with other people, we are only reinforcing this behaviour*” (Mendes-Santos et al. (52), p. 8).

Finally, HCPs drew attention to the context of digital engagement itself, including the potential cognitive burden of DMHTs, which might contribute to cognitive overload and eliminate opportunities for mental respite. The use of smartphone-based apps was described as *“a missing break for the brain”* (Participant, Hildebrand et al. (46), p. 7), which suggests that constant digital engagement in everyday life might blur the boundaries between treatment and daily living and impede service users’ recoveries.

## Discussion

This systematic review is the first to comprehensively synthesise qualitative data exploring HCP views and/or experiences of DMHTs across a range of clinical healthcare settings. The aims of the review were fully met because we enhanced our understanding of HCP perspectives regarding the use of DMHTs in clinical settings.

A central finding across all synthesised studies was the shared view that DMHTs should not be used as a replacement for face-to-face clinical care. This finding reinforces existing literature that DMHTs should be used alongside face-to-face interactions in a way that preserves the *‘human connection’* (16, 63). Wosny et al. (26) identified mixed attitudes among HCPs towards digital interventions in physical healthcare, and Eisner et al. (24) identified certain clinician attitudes (e.g., that there was no benefit to DMHTs/DMHTs would replace face-to-face care) might be a barrier to their implementation in mental healthcare. Our review extended these findings; for example, the nuance in HCPs’ language (e.g., *“indispensability of face-to-face contact”*) added a layer of qualitative richness not widely documented before.

Furthermore, our review added new depth by revealing that HCP attitudes were not uniformly resistant; rather, they were shaped by the perceived role of DMHTs within care pathways. HCPs in this review were open to DMHTs as adjuncts to care, which supports previous calls for hybrid and/or stepped-care models (64, 65). HCPs in this review also recognised specific benefits of DMHTs in enhancing assessment accuracy, symptom tracking, and care accessibility, which aligns with previous research by Luxton et al. (66) who suggested DMHTs offered greater accuracy compared to retrospective symptom reporting and might therefore enhance mental health treatment options.

HCPs in our review highlighted several benefits of DMHTs for service users (e.g., improved access, stigma reduction and empowerment). Whilst stigma reduction and empowerment had been cited as benefits of DMHTs for service users in previous studies (67, 68), our review deepened the narrative by exploring *how* HCPs perceived these benefits in different service user contexts. For example, HCPs noticed that DMHTs might have been particularly helpful for individuals who experienced barriers to traditional, face-to-face help-seeking, which adds weight to the argument that DMHTs play a unique role in engaging hard-to-reach populations (69, 70).

Nonetheless, this review also emphasised the nuance in HCPs’ perceptions of their appropriateness for different service user populations (e.g., age, digital literacy and socioeconomic status). These perceptions align with prior research into the “digital divide”, in that disadvantaged populations might struggle to access or benefit from digital tools (57, 71). However, some of these findings might reflect HCPs’ misperceptions; for example, some studies have shown that older individuals demonstrate much better engagement with online interventions than younger participants (e.g (72, 73)).

The current review also indicated that HCPs might struggle to ensure specific digital exclusions do not exacerbate existing health inequalities. Other concerns were clearly identified. HCPs expressed hesitancy about using DMHTs with high-risk service users (e.g., those experiencing suicidality), despite previous evidence of positive outcomes in these groups (74, 75). HCPs hesitance may reflect the relatively new and evolving nature of digital tools in clinical practice (76, 77) and the lack of established safety protocols for service users presenting with significant risk.

Other concerns highlighted by HCPs in this review (e.g., workload burden, risk management concerns, and risk of harm to service users) parallel existing implementation science literature which suggests that new innovations (such as DMHTs) often fail due to poor integration with existing systems (78) and concerns over risk and liability (23, 79). HCPs in this review considered that DMHTs might carry a risk of harm to service users (e.g., increased anxiety/focus on symptoms), which parallel previous review findings by Allan et al. (80) who reported that mental health exacerbations have been flagged as adverse events for some digital interventions. As previous research has suggested that there is inconsistent reporting across digital health interventions (81), our findings further underscore an emergent need for clear safety measures to support safe implementation of DMHTs in clinical services. With regards to workload burden, previous studies have also identified workload pressure as a barrier to implementation (21, 24, 82); however, our review offers a rich contextual understanding of HCPs’ experiences, specifically the legal and emotional weight of managing risk alerts via DMHTs, which had not been reported in the literature before.

### Clinical implications

Findings from this systematic review have several implications for clinical practice, with specific implications across stakeholder groups illustrated in **Table 6**.

**Table 6:** Clinical implications of DMHT implementation across stakeholder groups.

Stakeholder group	Specific implications
Service users	-Service users’ digital literacy and delivery format preferences must be considered during the initial assessment. -Service users’ experiences of DMHT use as part of their care should be reviewed regularly. Any adaptations/changes should be made based on service user feedback. -DMHTs might be particularly useful as early intervention tools and/or within a stepped care model, namely for service users with low intensity/less severe mental health problems. -Hybrid models are acceptable, especially for users with low intensity needs – however – exclusively virtual models of care delivery should be avoided. -The risk of digital exclusion for certain service user groups should be considered and factored into service provisions.
HCPs	-HCPs should not use DMHTs as a replacement for face-to-face clinical care. -HCPs require digital training for effective delivery. -Protected time must be allocated within HCPs job plans for engaging with DMHTs (e.g., monitoring data, responding to alerts). -HCPs views and suggestions should be considered when establishing clinical risk protocols and responsibilities. -HCPs should be involved in the planning and decision-making around DMHT implementation to increase clinician buy-in.
Service leads	-Service leads need to invest in appropriate infrastructure to support DMHT integration (e.g., IT systems, training frameworks). -Service leads need to commit to the facilitation of an organisational culture shift toward hybrid models of care. - Service leads should support frontline HCPs to develop co-produced risk management protocols. - Service leads must ensure adequate resource allocation for protected time and supervision for HCPs delivering DMHTs.
Commissioners	- Commissioning strategies should support blended care models that preserve face-to-face treatment for those with complex needs. -Commissioners’ investment in infrastructure and workforce development is critical to implementation. -Commissioners need to prioritise digital equity, ensuring access for underserved populations. -Commissioners should support the ongoing evaluation and quality assurance of commissioned DMHTs in clinical services.

Our review suggests DMHTs should be framed and implemented as tools that enhance, but do not replace, face-to-face care. Stakeholders should work to integrate DMHTs within existing clinical workflows and frameworks, rather than positioning DMHTs as stand-alone treatment options. Participant narratives indicated that DMHTs might be particularly effective when used as part of a stepped model of care, for example, as an early intervention/prevention tool. In addition, findings from this review suggest that using DMHTs as part of a hybrid model of care is acceptable, particularly for service users with low intensity difficulties. Integrating DMHTs in this manner might offer a solution to the growing demand on mental health services (83–85) whilst preserving face-to-face treatment for service users with more severe and complex mental health presentations.

Findings from this review have underscored the importance of clinical discretion and promoting service user autonomy in terms of digital treatment planning (85, 86). In clinical practice, this could be adopted by integrating questions about service users’ digital literacy and intervention delivery format preferences within initial assessment/triage appointments. This would ensure that DMHT prescribing is personalised to the service user on a case-by-case basis.

However, concerns raised by HCPs in this review must be addressed prior to implementation. These include lack of clinician time, digital training needs, and unclear risk management protocols. This review has emphasised the infrastructure that is needed to scaffold DMHT implementation. For example, services must provide digital training to HCPs, as well as establish protected time within HCPs job plans to account for the additional responsibilities associated with monitoring, engaging with, and responding to DMHT-related data. This review has also highlighted the importance of co-produced protocols for handling clinical risk, with frontline staff involved in the decision-making process regarding DMHT implementation within their services (85). Without all of this, there is a risk that DMHTs will exacerbate existing service pressures rather than alleviate them.

### Strengths, limitations and future research

This systematic review contributes a novel synthesis of qualitative evidence exploring HCPs’ views and/or experiences of DMHTs. A comprehensive systematic search of peer-reviewed and published literature was conducted, and data were synthesised from 15 studies reflecting the voices of 604 HCPs across ten countries and spanning 13 years of research. A range of professions, healthcare settings and digital tools were represented in HCP samples, which supported an in-depth analysis and interpretation. Whilst prior reviews have largely focused on digital health tools in physical healthcare or on specific modalities, such as passive sensing (27), this review uniquely captured HCPs’ understanding of the contextual, interpersonal and ethical considerations involved in DMHT use in mental healthcare. Furthermore, the use of thematic synthesis was a particular strength because this supported the development of a rich understanding of how DMHTs were perceived by HCPs, and it offered new insights into factors influencing uptake within clinical services. Another key strength was the methodological rigour of the review, with its transparent and systematic approach to data collection, quality appraisal and synthesis of studies. Consideration of reflexivity as well as adherence to ENTREQ guidelines (30) further strengthened the transparency of findings.

Several limitations are also acknowledged. Firstly, only English-language studies were included, which might have introduced location bias and excluded valuable and relevant perspectives from non-English-speaking HCPs. Secondly, this review was limited to peer-reviewed literature only. Whilst this increased the methodological quality of this review, it is possible that this decision might have introduced publication bias because grey literature and non-academic sources were excluded (87). Thirdly, albeit with a substantial agreement of 99.75%, only 10% of the studies were subjected to dual independent screening, which means that we cannot rule out potential selection bias of study inclusion. The rigour and trustworthiness of the review findings could also have been strengthened by including a second reviewer in the analysis of all included studies. Fourthly, as our search terms focussed on digital mental health tools more generally and did not use the term ‘digital therapeutics (DTx)’, it is possible that some relevant studies could have been missed.

Fifthly, whilst reviewed studies included a range of HCPs, only 60% of studies (*n* = 9) specified full details for the job roles of staff, client population served and service setting/context, which limited our ability to fully explore profession- and setting-specific perspectives on DMHTs. Most studies included in this review discussed DMHTs in broader terms, and only 26.7% of studies (*n* = 4) included HCPs views on *specific* digital tools that had already been implemented within clinical settings. We deliberately used a relatively broad definition of DMHTs and did not list specific categories, such as telepsychiatry platforms, clinician-facing decision-support tools, patient portals or open notes, because this approach could have limited the number of studies we were able to identify. Furthermore, we aimed to explore the views HCPS had about DMHTs more generally. As DMHTs represent a rapidly evolving field, future research might concentrate on studies that target specific DMHTS or those DMHTs currently being used within mental health care settings. It would also be useful to conduct an integrated systematic review in future in which HCPs’ concerns (e.g., guided versus unguided online interventions, familiarity with technology, etc.) are reviewed against existing quantitative evidence.

## Conclusion

This was the first review to comprehensively synthesise qualitative research exploring HCPs’ views and/or experiences of DMHTs in clinical practice. Whilst HCPs recognised the potential for DMHTs to enhance and increase access to mental health care, concerns remained in relation to therapeutic quality, risk management and workload. We recommend context-sensitive implementation that is supported by appropriate infrastructure and tailored to individual needs.

## Data Availability

The original contributions presented in the study are included in the article/supplementary material. Further inquiries can be directed to the corresponding author.
